# Spike-Timing Dependence of Structural Plasticity Explains Cooperative Synapse Formation in the Neocortex

**DOI:** 10.1371/journal.pcbi.1002689

**Published:** 2012-09-20

**Authors:** Moritz Deger, Moritz Helias, Stefan Rotter, Markus Diesmann

**Affiliations:** 1 Bernstein Center Freiburg, Albert-Ludwig University, Freiburg, Germany; 2 Computational and Systems Neuroscience, Institute of Neuroscience and Medicine (INM-6), Research Center Jülich, Jülich, Germany; 3 Faculty of Biology, Albert-Ludwig University, Freiburg, Germany; 4 Medical Faculty, RWTH Aachen University, Aachen, Germany; Indiana University, United States of America

## Abstract

Structural plasticity governs the long-term development of synaptic connections in the neocortex. While the underlying processes at the synapses are not fully understood, there is strong evidence that a process of random, independent formation and pruning of excitatory synapses can be ruled out. Instead, there must be some cooperation between the synaptic contacts connecting a single pre- and postsynaptic neuron pair. So far, the mechanism of cooperation is not known. Here we demonstrate that local correlation detection at the postsynaptic dendritic spine suffices to explain the synaptic cooperation effect, without assuming any hypothetical direct interaction pathway between the synaptic contacts. Candidate biomolecular mechanisms for dendritic correlation detection have been identified previously, as well as for structural plasticity based thereon. By analyzing and fitting of a simple model, we show that spike-timing correlation dependent structural plasticity, without additional mechanisms of cross-synapse interaction, can reproduce the experimentally observed distributions of numbers of synaptic contacts between pairs of neurons in the neocortex. Furthermore, the model yields a first explanation for the existence of both transient and persistent dendritic spines and allows to make predictions for future experiments.

## Author Summary

Structural plasticity has been observed even in the adult mammalian neocortex – in seemingly static neuronal circuits structural remodeling is continuously at work. Still, it has been shown that the connection patterns between pairs of neurons are not random. In contrast, there is evidence that the synaptic contacts between a pair of neurons cooperate: several experimental studies report either zero or about 3–6 synapses between neuron pairs. The mechanism by which the synapses cooperate, however, has not yet been identified. Here we propose a model for structural plasticity that relies on local processes at the dendritic spine. We combine and extend the previous models and determine the equilibrium probability distribution of synaptic contact numbers of the model. By optimizing the parameters numerically for each of three reference datasets, we obtain equilibrium contact number distributions that fit the references very well. We conclude that the local dendritic mechanisms that we assume suffice to explain the cooperative synapse formation in the neocortex.

## Introduction

The structure of neocortical networks of neurons changes in time: new synapses are formed, maturate, and eventually are pruned again, in the adult as well as in the developing animal [1], [2], for recent reviews see [3], [4], [5]. The majority (about 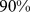) of excitatory synaptic contacts terminate on dendritic spines [6], and dendritic spines almost always (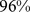) form a synapse [7]. The synapses on dendritic spines are highly dynamic [8], [9], for example [10] found an average spine turnover of 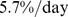 in primary visual cortex and of 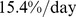 in somatosensory cortex. Yet in the adult animal, the statistics of the numbers of synapses are preserved over time, indicating that synapse creation and pruning balance each other [11], [12], [13]. According to theoretical studies on associative networks, structural plasticity enhances the memory capacity of a network substantially [14], [15], and has been shown to be related to motor learning in the brain [16].

The three studies [17], [18], [19] reported the distributions of numbers of synaptic contacts for different intra-cortical synapses in rat somatosensory cortex. Fares et al. [20] subsequently analyzed whether the reported distributions could result from random and independent synaptic contact formation, given a set of potential sites (close appositions) between axons and dendrites of reconstructed cells. As they showed, independent formation of synaptic contacts alone cannot explain the distributions. In addition a cooperative pruning mechanism, by which synaptic contacts that constitute a single synapse stabilize each other, is required to explain the observed distributions.

Here we build on grounds of this work and go beyond it in two aspects: Primarily, we consider synaptic processes that operate continuously in time. Secondly, we investigate an explicit candidate mechanism for the cooperation between synaptic contacts: Local correlation detection at the dendritic spines and thus dependent pruning and maturation of spines.

Recently Kasai et al. [21] summarized known properties of the plasticity of dendritic spines. Their model [22] describes the dynamics of the volume of dendritic spines. Here we restrict this model to three distinct categories of synapse states and introduce an explicit spike-timing dependence. Other models of structural plasticity [23], [24], [25] are based on the firing rate of the neurons. Consequently, in these models spike-timing and correlations of the spiking activity do not play a role, so they cannot show the mechanism of synaptic cooperation that we hypothesize here. The relative timing of pre- and postsynaptic activity indeed influences structural plasticity at the dendritic spine [26]. In contrast to previous models, the model of Helias et al. [27] is sensitive to the spike-timing of the pre- and the postsynaptic cell and describes structural plasticity in biophysical terms of protein kinetics in response to synaptic input. Here we choose an intermediate scale by still describing single synaptic contacts, but with a higher level of abstraction than previous work [22], [27]. The goal of the present work is to demonstrate the potential of local correlation detection at the spine, while making minimal assumptions about the involved biophysical processes. The assumptions entering our model, as introduced in detail in Methods, required to qualitatively explain the experimental results, are: a) presynaptic release of glutamate causes postsynaptic depolarization at excitatory synapses, b) depolarization electrically spreads within the dendrite, c) there is a correlation sensing mechanism sensitive to the relative time of presynaptic and postsynaptic firing (e.g. NMDA receptors) that causes downstream effects on the evoked synaptic amplitude in a spike timing dependent plasticity (STDP, [28]) like manner, d) synapses with small amplitude are more likely to be pruned than strong ones. Because of its analytical tractability, we can compute the steady state of our model and match its parameters to experimental reference data, analogous to Fares et al. [20]. Our results show that no direct signaling between synaptic contacts is necessary to explain cooperative synapse formation. In contrast, it suffices that distinct synaptic contacts cooperate in exciting the postsynaptic neuron, and thereby indirectly affect spike-timing dependent structural plasticity at other synaptic contacts.

## Methods

In this section we introduce a model of structural plasticity and describe the optimization procedure to fit the model to the experimental reference data.

### Correlation trace at the synaptic contact

Let us first introduce a simple model for the correlation detection at the postsynaptic dendritic spine. An action potential of the postsynaptic neuron causes a depolarization at the site of each dendritic spine. The spine has the biophysical substrate to maintain a signal that depends on the time of the action potential in relation to the time when a presynaptic impulse arrived [27]. Here, we call this signal the correlation trace 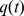 and assume a phenomenological model: if the presynaptic neuron spiked shortly before the postsynaptic one, the correlation trace is increased by 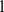. We call this a causal event. For the opposite relative timing, called an anti-causal event, the trace is decreased by 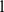. The trace therefore counts causal and anti-causal combinations of pre- and postsynaptic spikes. Further we assume that the correlation trace is forgetful: it has a leak with the time constant 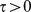, and we also assume that there is some additive noise in the process. The dynamics of the correlation trace at the synapse is given by the stochastic differential equation [29]

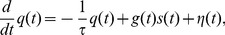
(1)where 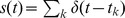 is the spike train of the postsynaptic neuron, with the spike times 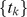, the factor 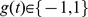 specifies if the particular spike is counted as a causal or anti-causal event, and 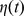 is an additional white noise with mean zero 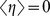 and infinitesimal variance 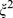. Mathematically, the trace 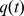 is identical to a shot noise [30] with the exponential kernel 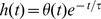 driven by the stochastic input process 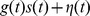.

Let us now introduce a minimal model of correlated spiking of the neurons. For each postsynaptic spike, we speak of a causal event at a given synaptic contact if the closest spike of the presynaptic neuron occurred prior to the postsynaptic one (because it could have caused the postsynaptic spike). If the closest presynaptic spike occurred after the postsynaptic one, the event is called anti-causal. Suppose that the probability for a causal event is given by 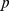. If both of the neurons fire independently, then 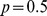. We define 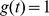 with probability 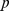 and 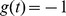 with probability 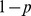 for each postsynaptic spike in 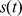.

Strictly speaking, the process defined by (1) is unphysical, since through 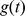 it depends on events in the near future (because the time of the next presynaptic spike has to be known). We hence consider (1) as an effective, adiabatic description of the correlation trace, since we are only interested in the statistics of the trace on long timescales. A process like (1) could result from several biophysical implementations that do in fact respect causality. For example the synaptic weights in phenomenological models of spike timing dependent plasticity, for which causal implementation are known [31] follow dynamics similar to (1). An example of a cellular mechanism to implement (1) is the number of activated CaMKII macro-molecules [27] or long-term potentiation [32], [33], [34].

Now let us further assume that postsynaptic spikes 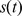 occur according to a Poisson point process with rate 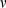. The firing rate 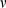 comes about through integration of thousands of synaptic inputs, and the particular synaptic connection modeled here only provides a small contribution to 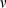. Since structural plasticity is known to be a slow process compared to the activity of neurons, and since the time constant of possible candidate mechanisms for the correlation trace can be considerable [35], a large integration time constant 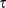 is reasonable, such that 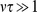. Then the equilibrium probability distribution of 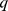 is a normal distribution with mean 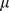 and variance 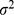,
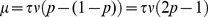
(2)

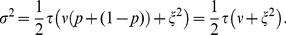
(3)
Eqs. (2, 3) can be obtained by considering two independent stochastic processes 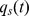 and 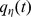 with 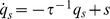 and 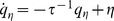. Then 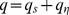 and mean and variance of 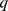 follow from summing the respective statistics of 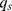 and 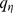, which can be obtained using standard techniques [30]. Note that 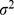 is independent of 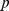.

The probability of causal spike pairings 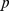 depends on the number of active synapses 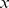 connecting the presynaptic neuron to the postsynaptic one, because each excitatory synapse increases the chance of the presynaptic neuron to make the postsynaptic neuron fire. As demonstrated for integrate-and-fire neurons in [36], the probability of a spiking response to a presynaptic spike is proportional to the synaptic weight of the input spike for a wide range of magnitudes of the synaptic strength. If the membrane potential of the postsynaptic neuron integrates the inputs linearly, the synaptic weight of the input from the presynaptic neuron is proportional to the number of active synaptic contacts between the neurons. So the probability of a spike response, and so of a causal event, rises proportionally to the number of active synaptic contacts. Effectively we thus assume 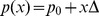, where 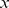 is the number of active synaptic contacts between the presynaptic to the postsynaptic neuron, 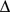 is the response probability per synapse, and 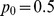. The two components of the probability 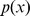 can be interpreted as the probability 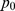 for a causal event by chance due to the Poisson firing of the postsynaptic neuron with rate 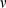 and the probability 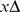 exceeding chance level triggered by the arrival of the presynaptic spike.

In order to obtain an estimate of 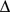 consider a single synaptic contact between two neurons that, upon activation, causes an excitatory postsynaptic potential (EPSP) with amplitude 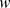. For a leaky integrate-and-fire model neuron in the asynchronous state [37] resembling cortical activity, we can read off the response probability 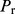 of a neuron to such a voltage jump from Fig. 4D in [36]. So we have 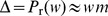 with 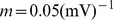. The respective values of the EPSP size 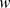 per contact have been reported along with the reference datasets in [17], [18], [19], and we list them among the model parameters in Tab. 1. We thus arrive at the estimate for the probability of a correlated pairing
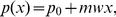
(4)and thus with (2) the mean of the stationary distribution of 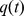 is
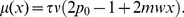
(5)Note that the linear model (4) for the probability of causal spike pairings may for large 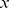 and 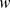 yield values of 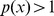, which are nonsensical. A consistent definition of 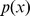 should saturate when reaching the value 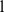. Taking into account this saturation at large 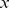, however, would not make a difference for the models considered here, because all solutions for synapse distributions found below exhibit vanishing probably throughout at such large values of 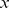.

**Table 1:** Reference data, optimized parameter sets and properties of the structural plasticity models.

Connection	L4-L2/3	L5-L5		L4-L4	
Reference	[17]	[18]		[19]	
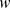 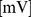	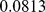	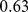		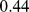	
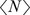 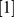	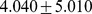	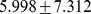		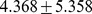	
displayed in	Fig. 1a	Fig. 1b	Fig. 2a	Fig. 1c	Fig. 2b
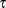 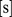	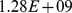	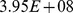	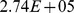	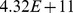	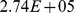
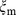 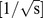	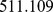	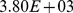	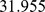	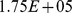	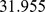
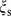 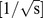	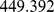	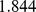	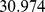	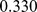	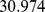
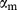 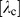	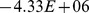	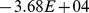	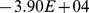	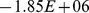	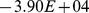
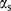 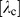	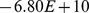	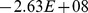	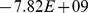	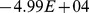	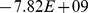
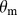 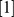	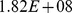	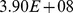	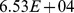	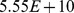	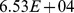
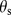 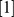	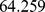	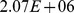	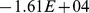	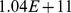	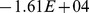
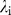 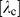	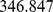	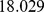	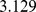	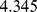	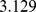
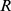 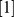	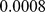	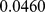	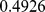	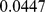	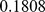
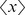 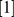	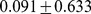	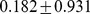	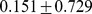	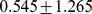	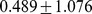
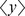 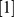	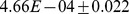	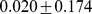	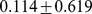	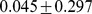	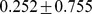
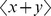 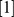	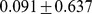	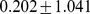	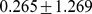	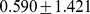	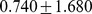
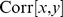 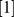	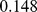	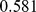	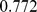	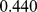	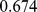
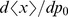 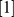	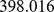	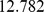	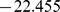	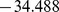	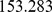
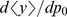 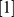	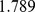	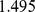	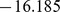	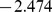	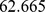
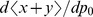 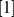	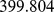	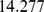	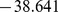	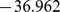	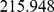
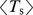 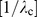	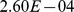	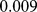	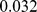	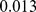	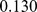
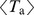 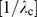	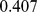	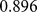	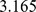	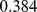	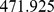
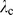 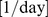	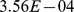	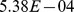	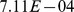	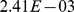	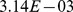
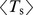 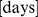	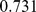	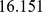	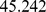	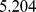	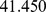
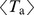 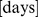	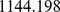	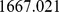	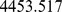	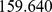	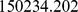

**Figure 1:**
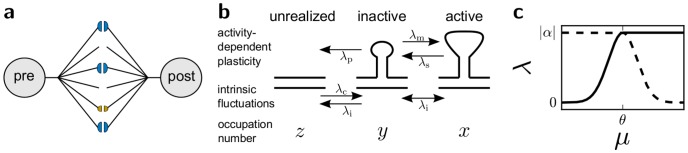
Illustrations. ***a***: Schematic of a synapse connecting a pair of cortical neurons (pre- and postsynaptic). Individual synaptic contacts can be active (blue, larger; corresponding to a large dendritic spine), inactive (orange, smaller; corresponding to a thin dendritic spine) or unrealized. ***b***: State diagram of the structural plasticity model adapted from [22]. A synaptic contact can occupy one of three states: active, inactive or unrealized. Transitions from one state to the other are possible through either intrinsic fluctuations or activity-dependent plasticity, indicated by the arrows. The transitions from unrealized to inactive are called “creation” (rate 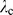), from inactive to active “maturation” (rates 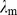 and 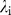). The transitions from active to inactive are called “shrinkage” (rates 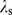 and 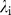) and from inactive to unrealized “pruning” (rates 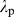 and 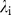). A synapse consists of 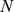 close appositions which may or may not host inactive or active synaptic contacts. The occupation number of the three states, active, inactive, unrealized are called 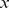, 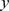 and 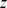, respectively. ***c***: Assumed functional form of the dependence of the rate of activity dependent transitions (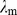, 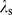, 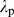) on the mean value 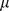 of the correlation trace, which is formed at the dendritic spine. The absolute value of 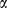 sets the scale of the transition rate, while its sign flips the function from right-saturating (solid) to left-saturating (dashed). The parameter 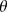 determines at what correlation level saturation of the transition rate is reached.

Description of listed values top to bottom: Section 1) Connection properties: Unitary EPSP amplitude 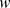 per contact as published in the reference, expected number of close appositions 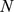 from [20] and standard deviation of 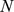, figure in which properties of the model with this parameter set are displayed; Section 2) Model parameters: Correlation trace time constant 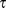 (1), amplitude 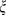 of correlation trace noise, scale 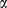 of transition rates (see eq. 7 and Fig. 1***c***), saturation threshold 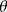, intrinsic maturation/shrinkage/pruning rate 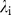; Section 3) Model properties: Sum of squared residuals 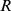 of the model (see eq. 19), expected numbers of active (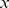), inactive (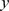) and total (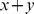) synaptic contacts and standard deviation of 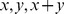, Pearson correlation coefficient of 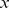 and 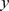, derivatives of expectation of 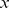, 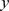 and 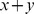 with respect to the probability of causal spike pairings (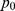) (estimated numerically), expected lifetimes of inactive and active contacts (see eq. 23 and eq. 24), estimate of model time scale 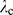 to match physiological spine turnover ratio (25), lifetimes in units of days using 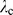 estimate. Additional, fixed parameters that are common to all models are 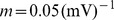, 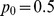 and 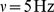.

So far, we hypothesized a generic correlation detection mechanism at each synaptic contact and computed its equilibrium statistics (5) and (3) for multiple excitatory contacts between two neurons. In our model the values of the correlation trace of each synaptic contact follow a normal distribution, specified by the its mean 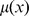 and variance 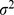, which depend on the parameters 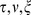. Note that only the mean 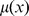 depends on the number 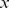 of active contacts, whereas 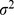 is a constant. To reduce the amount of free parameters of the model, we further set 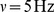 which is a reasonable choice for neocortical neurons.

### Structural plasticity based on the synaptic correlation trace

The synaptic correlation trace can guide structural plasticity. Because of a lack of detailed knowledge about the biomolecular mechanisms involved [38] we again employ a simple effective model. As structural plasticity is a slow process compared to the spiking activity of neurons, we assume that the distribution of the correlation trace at each synaptic contact is effectively in stochastic equilibrium throughout. This is also known as an adiabatic approximation. Let us now assume that a structural change of the synaptic contact is initiated when the correlation trace crosses a boundary value 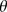. A biochemical mechanism underlying this assumption could be the activation of a signaling pathway when a specific number of activated CaMKII molecules is reached [39]. Given the random trajectory of the correlation trace 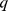, we need to know how long it takes until 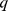 crosses the boundary upon which the synaptic contact makes the transition. This is known as a first passage time problem with an absorbing boundary. The inverse of the mean first passage time is called the escape rate. For simplicity, let us approximate 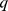 as a Brownian motion with the same infinitesimal mean and variance as the actual process (1). Then, according to the Arrhenius approximation [40], the escape rate is
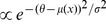
(6)in the case that the values of 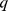 are far from the boundary 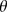, such that 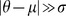. Here the proportionality constant is called the Arrhenius constant. Now what happens to the rate of structural changes when 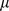 approaches 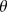
? If we take the model of a boundary crossing process seriously, then the escape rate should diverge as 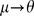. However, in a biological system it is more plausible that the rate of structural changes converges to a certain maximum rate which the cellular machinery can achieve. Based on this argument we construct our model for the rate of structural changes by extrapolating from the Arrhenius approximation (6), forcing it to eventually converge to a plateau,
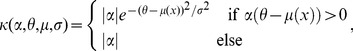
(7)with 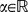. Depending on the sign of 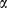, 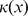 either approaches or departs from the plateau for increasing 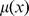.

As our assumptions about the biophysical implementation are quite general, they can model maturation, shrinkage and pruning of a synaptic contact alike. However, these are distinct processes that take place during different stages in the life cycle of a synaptic contact. For example, we cannot assume that the correlation detector noise 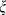 has the same magnitude for small and large dendritic spines since the number of channels mediating the signal might be different for the two. Therefore we use the model (7) for maturation, shrinkage and pruning, but choose a different set of transition parameters 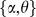 and correlation trace noise 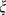 for each case. We decorate quantities associated to maturation with 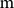, those associated to shrinkage with 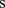 and pruning with 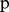. So the rate of maturation transitions is defined as
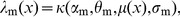
(8)the rate of shrinkage transitions as
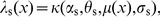
(9)and the rate of pruning as
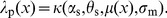
(10)The correlation trace parameters 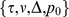 are assumed to be identical for both thin spines (inactive synaptic contacts) and large spines (active synaptic contacts), which will be defined below. The pruning rate uses the same parameters as shrinkage, except for the noise magnitude 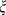 of maturation, because pruning is assumed to take place in thin spines.

Apart from the activity dependent transitions, the model also includes intrinsic fluctuations as in [22], see Fig. 1B. We assume that random maturation (enlargement), shrinkage and pruning of a spine occurs constantly with the rate 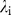, and the creation of new thin spines with rate 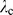.

Let us summarize the structural plasticity model we have introduced. At each of the synaptic contacts between a pair of neurons, a correlation trace is formed by counting causal and anti-causal pre-post spike pairings. The distribution of the values of the correlation trace depends on the number of active synaptic contacts since they all contribute to firing the postsynaptic neuron. We have further assumed that the activity-dependent structural changes of synaptic contacts depend on the correlation traces. Finally we also included intrinsic fluctuations of the synapse configuration.

### Steady state of the synapse

Above we defined a model for structural plasticity for the synapse between a pre- and a postsynaptic neuron. Although in this model the individual synaptic contacts may continuously change, the state of the synapse develops towards a stable steady state. A synapse typically consists of many individual synaptic contacts, as depicted in Fig. 1A. The neocortex is densely packed with only very limited unoccupied extracellular space. Accordingly, pairs of neurons cannot form arbitrary numbers of synaptic contacts [41]. Fares et al. [20] investigated reconstructed cortical tissue and counted the numbers of close appositions between pairs of neurons. At such a close apposition a synaptic contact may form, but is not necessarily present. Describing these results statistically, a probability distribution for the number of close appositions 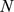 between two neurons can be obtained [20].

At each of the close appositions, the neurons may form a synaptic contact; in our model, we treat the different volumes of spines and EPSP amplitudes in a coarse-grained fashion, distinguishing just three different states for each contact to occupy – active, inactive, or unrealized. An active synaptic contact here describes a larger dendritic spine that contains both AMPA and NMDA receptors. An inactive contact models a thin, either newly formed or recently shrunk dendritic spine that has much less AMPA receptors [42], [43] and contributes little to firing the postsynaptic neuron. An unrealized contact, finally, is a close apposition where no contact has formed, but might be formed in the future. It is a close apposition without an established synaptic contact and corresponds to the potential synapse in [20]. A similar model has previously been proposed in the context of associative networks [44], [45].

We denote the numbers of synaptic contacts in these three states by 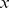, 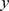 and 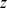 respectively. Since at any time 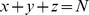, the state of a synapse is unambiguously defined by the combination of the number of active and inactive contacts 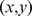. Now consider an ensemble of independent synapses, each with the maximum contact number 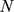. The probability 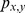 of a synapse to be in the state 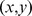 evolves in time according to the Master equation [46]

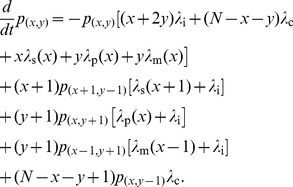
(11)The first term sums up the rate of leaving the state 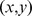 by all possible transitions. The second and third terms sum up all possibilities to go into state 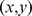 from other states by shrinkage and pruning, and the fourth term by maturation. The last term considers the transitions due to the creation of inactive synapses, the rate of which is given by 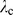 (see also Fig. 1B). The steady state distribution does not depend on the time scale of the transition rates, so we can consider the constants 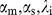 in units of 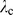. The time scale of the structural plasticity is then set by 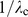. In (25) below we will see how the value of 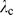 can be determined by experimental data.

To determine the steady state configuration of the synapse, let us introduce a numbering of all the possible synaptic states 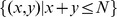, such that the probability of each state 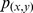 is represented by the value 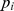, with a one to one correspondence between indices 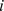 and states 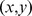. Then (11) can be written as
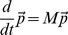
(12)where the entries of the matrix 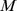 can be read off the Master equation. Since 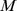 describes a Markov process it is column-stochastic (which means all columns sum up to zero). Since the process is irreducible, according to the Perron-Frobenius theorem there is only one stationary solution. We can determine the stationary probability distribution 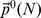 by solving 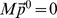 under the constraint that 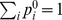. We implemented the construction of the matrix 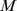 efficiently using Cython [47] and solved for the stationary solution using Scientific Python [48].

The stationary distribution depends on the number of close appositions 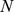. [20] have estimated the distribution 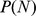 for the three types of intra-cortical connections that we consider. We incorporate this by determining 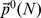 for each 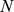 separately, and subsequently compute the averaged distribution
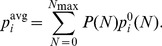
(13)Fares et al. [20] provide the distribution 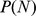 for 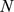 up to 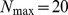.

For comparison with the reference datasets (see below), we are merely interested in the marginal probability of a certain total number 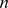 of synaptic contacts, disregarding whether they are active or inactive. The marginalization can be obtained from 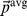 by summing over all states 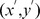 with 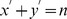, or more conveniently phrased as
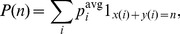
(14)where the function 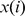 returns the value of 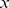 of the state 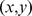 with index 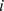, and 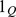 equals 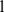 if 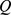 is true and 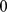 otherwise. Analogously, the marginal average distributions of the number of active and inactive synapses are
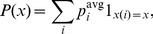
(15)

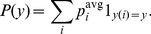
(16)


### Comparison to reference data and model optimization

**Figure 2:**
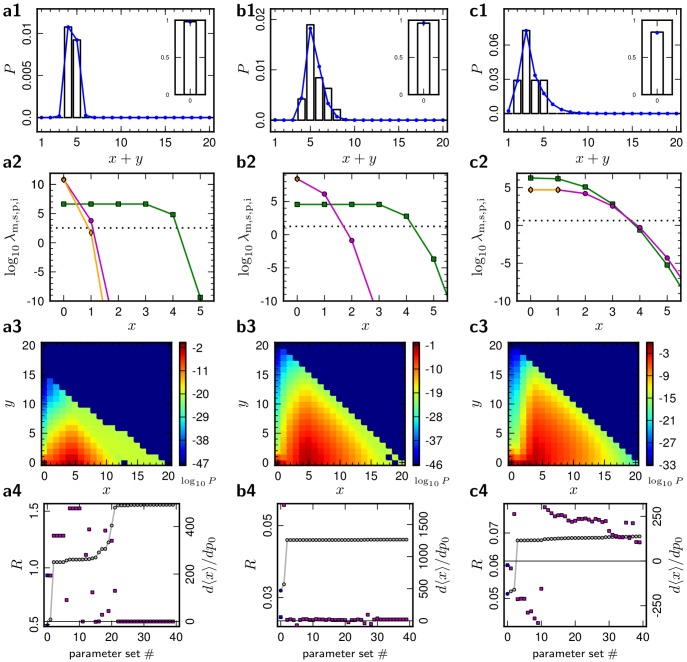
Single synaptic connection models. Best-fit individual models of the synaptic connections L4–L23 (***a***), L5-L5 (***b***) and L4-L4 (***c***). ***a1,b1,c1*** (row 1): Equilibrium distributions of the total number of synaptic contacts in the structural plasticity model (blue, eq. 14) compared to reference data (black). ***a2,b2,c2*** (row 2): Transition rate functions (7) of the models in the row 1, respectively, in units of 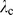. Activity dependent maturation rate 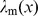 (green squares, eq. 8), shrinkage rate 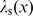 (orange diamonds, eq. 9), pruning rate 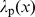 (magenta circles, eq. 10) and intrinsic maturation, shrinkage, and pruning rate 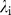 (dotted line). The spine creation rate 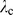 sets the time scale and has a value of 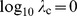. ***c1,c2,c3*** (row 3): Joint equilibrium distribution of the number of active (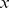) and inactive (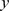) synapses. ***a4,b4,c4*** (row 4): Fit error 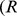, gray) and derivatives of active synaptic contact number with respect to baseline correlation (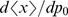, magenta) for the best 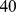 parameter sets, ordered by error 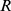. Blue markers indicate the model that is displayed above (rows 1 to 3). Model parameters and further information are given in Table 1.

In the experimental studies [17], [18], [19] a set of occurrence frequencies 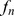 of numbers of synaptic contacts 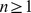 for several pairs of neurons was obtained, each for three different types of intra-cortical projections. Complementing this, for the same three datasets, the probability 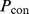 of a pair of neurons to be connected with at least one active contact can be estimated [20]. The distribution of the numbers of active synaptic contacts serve as reference data in our study. For each of the three datasets, we transform the reported data to the probability mass function
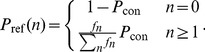
(17) we evaluate (14) and obtain the residuals
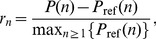
(18)for 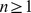. The residuals are scaled by the maximum of the reference distribution to enable comparison of the quality of the fits across reference datasets. We minimize the sum of squared residuals
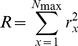
(19)using the Levenberg–Marquardt algorithm, applying its implementation from Scientific Python [48]. We call 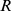 the error of the model. The optimization problem has several local minima, so we initialize the optimization procedure at many points in the 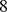-dimensional parameter space and compare the values of 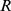 to which the optimization converged. Specifically, we choose four different initial values in each parameter dimension, which makes a total of 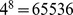 distinct optimization runs per reference dataset. The parameter sets which resulted in a minimal value of 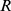 are shown in Tab. 1, along with additional information on the model, and the resulting equilibrium distributions are shown in Fig. 2.

**Figure 3:**
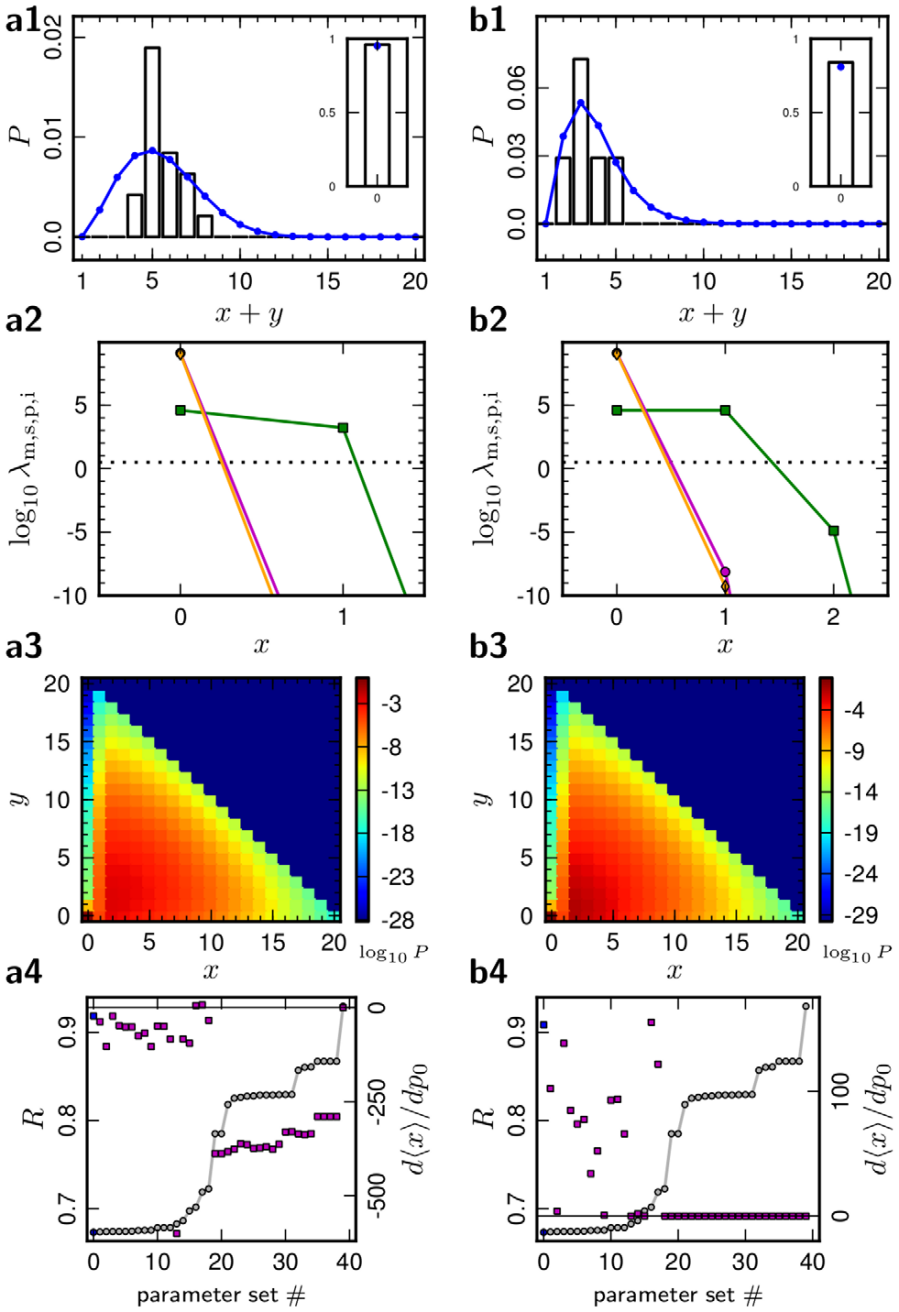
Model of two synaptic connections. Best-fit model for both synaptic connections L5-L5 (***a***) and L4-L4 (***b***) (same model parameters). ***a1,b1–a4,b4*** (rows 1 to 4) as in Fig. 2. Model parameters and further information are given in Table 1.

We also obtained parameter sets which yield good fits to two reference distributions simultaneously. The resulting distributions for the connections L4-L4 and L5-L5 are displayed in Fig. 3. Here the fit error was defined as the sum of the errors (19) of both distributions, 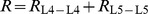. With respect to this the same optimization procedure was performed.

### Lifetime of synapses and turnover ratio

Here we compute the average lifetime of an inactive synaptic contact and of an active contact in the equilibrium state of the synapse model, see Fig. 1***a*** for the possible transitions. We define the lifetime 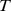 as the expected time until the contact is pruned. Consider an active contact in a synapse. It may become an inactive contact either through intrinsic or activity dependent shrinkage. The mean time up to the transition from active to inactive is 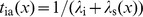. An inactive contact, on the other hand, might make a transition to the active state (maturation), which would take the time 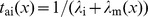, or to the unrealized state (pruning) in the time 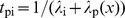. The mean time until the first transition, either maturation or pruning, is 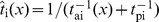. Either of the two transitions happens with a probability given by the fraction of rates involved, 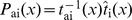 and analogously 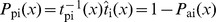. If the inactive contact becomes active, then it will become inactive eventually, and subsequently might be pruned or become active again. Accounting for the possible paths the inactive contact may take upon its first transition we obtain the expected lifetime of the inactive contact as
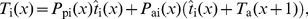
(20)where 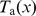 is the lifetime of an active contact in a synapse that has 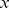 active contacts. In turn, starting from an active contact just adds one active to inactive transition, so
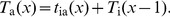
(21)Inserting (21) and the definitions above into (20) yields
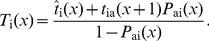
(22)We average the lifetimes across the equilibrium probability distribution of synapse states and obtain
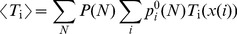
(23)

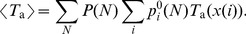
(24)


To match the time scale of structural development of our model to what is known from in-vivo studies we compute the spine turnover ratio as it is defined in [10],
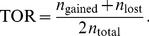
(25)Here 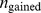 and 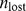 are the numbers of gained and lost spines during a given period of time, and 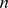 is the number of spines observed. In our model, the expectation values of these quantities are given as
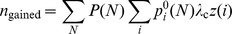


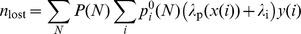


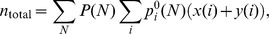
where 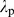 and 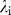 are given in units of 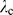. So we obtain 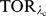 in units of 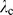 from (25). In rat somatosensory cortex [10] found 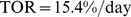. Accordingly (25) sets the time scale 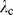 of the model to 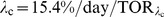.

### Necessity of inactive synapses for explanation of experimentally observed distributions of synaptic contacts

In this section we consider a simplified version of our model which does not include inactive synaptic contacts (thin spines). In that model, at a close apposition there can be either no synaptic contact or an active synaptic contact. Between these two states transitions are allowed just as between the inactive and the active state in the full model (see Fig. 1B), but here we call them 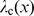 (creation) and 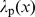 (pruning), which may yet be arbitrary functions. Assume there are 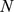 close apposition between a pair of neurons. Then the state of the synapse is defined by the number of active connections 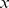. Let us denote the probability of the state 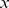 by 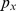 here. In stochastic equilibrium the probability fluxes into and out of the state 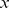 must balance, so for 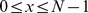 it must hold that
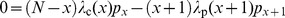
from which follows that
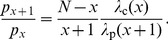
(26)Now consider the case of 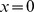. In all three reference datasets, 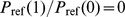, as can be seen in Fig. 2***a*** ***1–a3***. According to (26) this requires 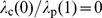, which can only be achieved by 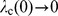 or 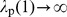. In contrast, around the secondary peak of the reference distributions 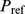, (26) entails that 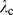 and 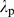 must be of comparable magnitude. More specifically, the right hand side of expression (26) has to change from values larger than 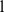 to values smaller than 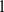 as 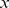 passes the secondary peak from below. To satisfy these requirements, even only approximately, demands highly non-monotonous choices of the functions 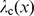 and 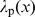 that are difficult to justify biophysically. Once the model includes the intermediate state of the inactive synaptic contact, however, it is possible to find biologically plausible parameter sets to explain the reference distributions, as described in the rest of the paper.

## Results

### Overview of the model

Kasai et al. [22] monitored the temporal evolution of the volume of dendritic spines and described it as a random walk with volume dependent drift and diffusion components. According to their findings, newly formed dendritic spines are small, and accumulate AMPA receptors as the spine volume increases. Thus a small spine can grow or disappear, and a large spine can shrink. Spines of all volumes, however, were found to contain NMDA receptors [42]. The study by Holtmaat et al. [10] suggests that thin spines are more readily pruned than thick spines, and that they may be of a lower efficacy or NMDA receptor-only (inactive) synapses. It was previously suggested 43,49 that small spines might correspond to silent synaptic contacts. In our model, we distinguish between only three states that each synaptic contact can occupy: active, inactive and unrealized, without considering the spine volume and channel density of the dendritic spines explicitly. These states correspond, respectively, to large spines, thin spines and close appositions with no spine, as illustrated in Fig. 1***b***. Note that we do not claim that the functional distinction between thin and large spines is actually as clear-cut as assumed in the model – the model merely represents a coarse-grained spine state. In this model, transitions between the three morphological/functional states are possible. Following [22] and [50], such transitions can occur either due to intrinsic fluctuations, or depending on the activity of the pre- and postsynaptic neuron. Note also that, although the inactive synaptic contact is allowed as a transitional state, it turns out to rarely occur in the optimized models that will be discussed below.

The basic idea of the model put forward in this study is the following: As described in [27] dendritic spines have the biomolecular capability of detecting correlations in the relative spike timing of the pre- and postsynaptic neuron. If there are several active excitatory synaptic contacts from a presynaptic neuron to a single postsynaptic cell, all these synaptic contacts contribute to elicit spikes in the postsynaptic neuron. Hence each of the contacts increases the correlation between the two cells, measurable at each of the corresponding dendritic spines. So even if there is no direct communication between the synaptic contacts, they affect each other indirectly by increasing the correlation of pre- and postsynaptic spikes. Spike-timing dependence of structural plasticity is thus a candidate mechanism for the cooperation between synaptic contacts.

According to [27] the magnitude of calcium influx into the dendritic spine depends on the proximity of pre- and postsynaptic spikes in time. The calcium influx activates or deactivates CaMKII macro-molecules and thus leaves a local memory. We call such a memory of the spike-timing correlation a correlation trace. The activation of the CaMKII subunits can be preserved for a long time [51]. The model we consider here, however, does not rely on the biophysical details of CaMKII activation, but just assumes a correlation trace is available. For the purpose of this study, the correlation trace could also come about by other mechanisms.

Employing this correlation trace, we introduce a phenomenological model for activity dependent maturation, shrinkage and pruning of spines depending on the correlation of the spike-timing of pre- and postsynaptic cell, as described in detail in Methods. The model is based on [22] and incorporates the basic properties of structural plasticity [21], activity-independent creation and pruning of spines, intrinsic fluctuations of spine volume, and activity-dependent spine remodeling. The set of all synaptic contacts connecting a given pair of neurons constitute a synapse, see also Fig. 1***a***. The state of a synapse is defined by the number of active contacts (large spines) 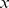 and inactive contacts (thin spines) 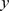. The time-evolution of the synapse state is then described as a Markov process. For a given parameter set, we solve for the stationary probability distribution of the states 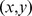.

### Fits to reference data

The parameters of the model were then optimized so that the distribution of the total number of synaptic contacts reproduces the experimental reference data, shown in Fig. 2***a1–c1***, along with the respective transition rates of the model (6) in ***a2–c2***. For each of the three reference datasets (***a***, ***b***, ***c***), we show the best model that resulted from the optimization. The models can reproduce the experimental distributions of synapse numbers.

The existence of such a stationary distribution means that the average numbers of inactive, active and potential sites of the synapse do not change in time. This is so despite the constant creation and pruning of synaptic contacts since these processes compensate each other in equilibrium. Implicitly, the model allows that inactive, active and potential sites coexist between a given pair of neurons.

The parameter sets for the displayed models are given in the second section of Tab. 1. The fit of the connection L4–L23 takes very different parameter values than the others. Nonetheless for all three modeled connections, the time constant 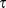 of the correlation trace is large compared to the time scale of fluctuations of neuronal activity, in agreement with our assumption about the distribution of the correlation trace. Concerning the parameters of the activity dependent structural plasticity, we find qualitatively similar results across datasets: In all three cases, both maturation and shrinkage/pruning rates decrease with increasing active synapse number, granting long-term stability to established synapses.

The models of the intralaminar connections L4-L4 and L5-L5 show remarkable similarities. Both have a comparable 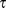 and the rate of intrinsic, activity independent transitions 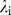 is low, although this was not an a priori assumption. The parameter values for 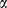 and 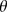 are difficult to interpret individually. Across all the models, inactive synaptic contacts are rare, as indicated by the fraction of 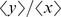 which ranges between and 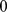 and 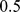. A fit of both connections L4-L4 and L5-L5 with a single parameter set is displayed in Fig. 3. Although the model distributions in Fig. 3***a1***,***b1*** do not follow the reference data as closely as in Fig. 2, a good agreement of the distributions and the reference is achieved.

### Properties of the optimized models

**Figure 4:**
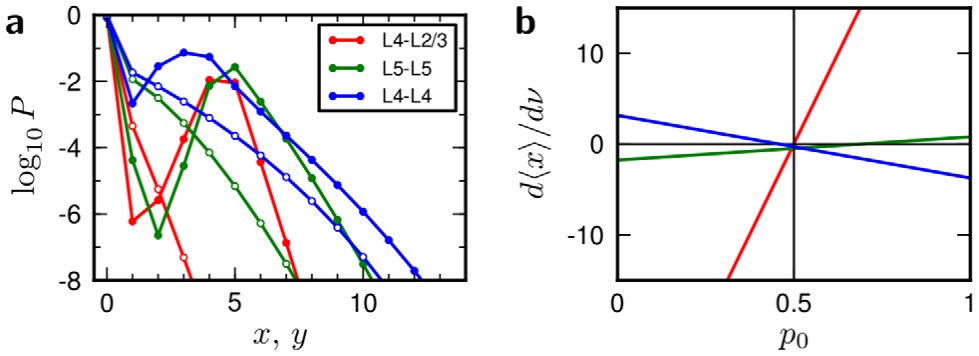
Further properties of the single connection models. ***a***: Marginal probabilities of active (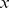, filled circles, eq. 15) and inactive (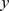, empty circles, eq. 16) synaptic contact numbers of the best-fit models shown in Fig. 1. ***b***: Derivative of the number of active synaptic contacts by the neuronal firing rate as a function of the baseline correlation 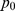 – values of the 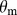 and 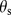 are shifted to keep the equilibrium distribution unchanged for all values of 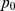 (27). Negative values indicate a stable equilibrium (firing rate homeostasis). Colors as in ***a***.

For each of the three reference datasets we obtained many models with a comparable fit error 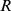. Fig. 2***a4–c4*** and Fig. 3***a4,b4*** show the error 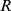 (circles) of the best parameter sets obtained, ordered by the value of 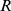. We also investigate how the model distribution changes in response to an increase in the baseline probability of causal spike pairing. Some models decrease their contact number, while other models increase it, as can be seen from the derivative 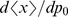 (squares in Fig. 3, 1, row 4). This quantity can take very different values for comparable fit errors 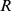. We call a model Hebbian if the number of active contacts grows upon an increase of causal spike pairings (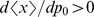). Conversely we call a model anti-Hebbian if the number of contacts decreases (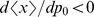). This diversity indicates that the plasticity model used here is general enough to implement Hebbian and anti-Hebbian learning, depending on the parameters. In Tab. 1 the value of the derivative 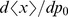 is given along with other properties of the selected models. For each dataset, we selected the best model irrespective of it being Hebbian or anti-Hebbian. Another characteristic property of a model is the joint distribution of active synapses 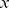 and inactive synapses 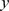 (Fig. 2 and 2, row 3). Especially in the model connections L4-L4 and L5-L5 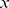 and 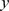 tend to be strongly correlated. While the expectation value of 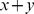 is largely determined by the reference data, the smaller expectation value of 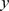 indicates that only a small proportion of spines are small and functionally weak. The marginal distribution of active and inactive synapses are shown for the three best models in Fig. 4***a***. In all selected models, across the datasets, the expectation values of 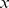 and 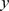 do not sum up to the expectation value of the number of contacts 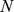, which means that many unrealized synapses (close appositions without inactive or active contacts) are present, consistent with experimental findings [21].

Fig. 4***b*** addresses the question whether a homeostasis of the neuronal firing rate can be achieved by this structural plasticity model. Here, a homeostasis means that an increase in firing rate leads to pruning of input synapses, thus lowering the firing rate in effect. Conversely a decrease in firing rate should lead to synapse maturation. If that is the case, the plasticity rule establishes a homeostatic control of the firing rate to a fixed value. In our model, a negative derivative 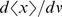 means that the plasticity rule acts as a firing rate homeostasis. From Eq. (5) we conclude that a change in 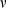 can either increase or decrease the value of 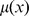 for a given 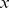, depending on the value of the baseline correlation 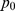. Previously, we arbitrarily set 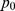 to 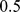. However, given a parameter set, we can change the value of 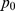 to 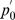 without changing the transition rates (and all equilibrium properties of the model) if we also shift the thresholds to
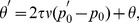
(27)because then all the distances 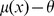 to the threshold are preserved. Thus 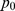 is effectively a free parameter of the model and can be adjusted to set 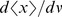, as is shown in Fig. 4***b***. Hence the structural plasticity model we propose can establish a firing rate homeostasis.

Furthermore we derived the expected lifetime of a synapse in the model, which is also shown in Tab. 1. Here the lifetime is defined as the expected time until the synapse is pruned. Before being pruned, it can go back and forth between the states inactive and active several times (fluctuate in volume). The lifetime is very different for inactive and for active synapses, the latter exceeding the former by about one order of magnitude or more. This is due to the fact that typically several active contacts coexist and mutually stabilize, which entails small rates 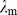, 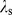 and 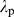 (cf. Fig. 2 row 2). If an active synapse becomes inactive, the rate to go back to the active state also increases, which promotes going back and forth through these states. This behavior matches nicely with the volume fluctuations of large dendritic spines described in [22]. Through (25) finally we can relate the time scale 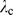 of the models to experimental data [10]. The values for 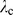 are listed in Tab. 1and confirm our assumption of a time scale separation of structural plasticity and neuronal activity. Using this estimate of the timescale, the lifetimes of inactive contacts are about a couple of days, while the lifetimes of active contacts span from a month up to years. [10] called spines with a lifetime of less than 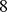 days transient, and spines with longer lifetimes persistent. This distinction roughly applies to the lifetimes of inactive and active contacts in our model.

## Discussion

We propose a model of structural plasticity to explain cooperative synapse formation [20]. The transitions of the states of synapses are assumed to depend on a signal locally available to a spine that depends on the correlation between pre- and postsynaptic activity, the correlation trace. There is strong evidence that a correlation trace could indeed be implemented in the dendritic spine through phosphorylation of the macromolecule CaMKII [35], [27], [34]. CaMKII has also been shown to be necessary for structural and long-term plasticity [52], [53], [4], and may also drive presynaptic changes [54]. Here we assume an abstract, effective correlation trace instead of explicitly modeling the dynamics of CaMKII. This makes our results independent of the specific mechanisms employed at the synaptic contact, since also other processes may be available to form the correlation trace. We assume the correlation trace at the spine is forgetful, such that it integrates causal and anti-causal spike pairing events like a leaky integrator with a certain time constant. This time constant affects the location of the equilibrium probability distribution of the correlation trace and its variance. Across the datasets L4-L4 and L5-L5, the time constants are comparable. If the correlation trace is implemented biologically by the cycle of expression, activation and degradation of CaMKII, these time constants will be observable in experiments. The optimized values for the time constant 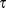 are well in the range of possible values that sustained CaMKII activation can show [51] for all three reference datasets. The differences in the model parameters of the connection L4–L23 compared to the other two intralaminar connections might be explained by the finding that most synaptic contacts of this connection are formed on dendritic shafts rather than on spines [55]. At dendritic shafts functionally similar plasiticity mechanisms could be at work, but our model might be less appropriate for this type of connection. However, although in early postnatal development more shaft synapses exist, in later stages synapses on spines dominate [56], [57].

The rates of structural changes at the synapse are assumed to be a function of the equilibrium correlation trace distribution. To model this dependence mathematically we chose a versatile functional form (7). This is necessary since a comprehensive quantitative description of the correlation dependence of structural plasticity is not known to date. Our optimization results for the transition rates show a strong selectivity for specific numbers of active contacts in a synapse: Transition rates are much higher in case there are few active contacts between two neurons, and many active contacts stabilize the system in all of the three modeled intra-cortical synapse types. Future experiments could investigate whether synaptic contact number (or EPSP amplitude) correlates with calcium transient amplitudes at the spines and with rates of spine maturation, shrinkage and pruning.

Using the optimized models we also computed the expected lifetimes synaptic contacts. The lifetime of active contacts is about ten to one hundred times larger than the lifetime of inactive contacts across our models. This can be understood given the experimental references' results that an active contact is always accompanied by several others. For such synapses, our models predict a vanishing rate of activity dependent transitions, which lets the synapses stay in the active state for a long time. Thus persistent spines here correspond to active contacts, and transient spines to inactive contacts. Our finding constitutes a statistical explanation of the existence of these two distinct classes of spines [10].

Our best-fit models show functional differences. Most notably, the models can be either Hebbian or anti-Hebbian, in the sense that an increase in the frequency of causal spike pairing leads to either increased or decreased numbers of active contacts. Both Hebbian and anti-Hebbian connections have been observed in the neocortex [58]. For all connections we found comparably good fits of both types. Furthermore our model predicts a joint probability distribution of active and inactive contacts which goes beyond current experimental references. Future experiments which determine both of these numbers for many neuron pairs will allow further evaluation of our model. A possibility to optically distinguish and monitor active and inactive synapses in experiments might be to use fluorescent markers for AMPA and NMDA receptors. Synaptic contacts that what we call “inactive” should show less AMPA than “active” ones, but the inactive ones also include those synapses with few AMPA receptors.

Previous models of structural plasticity have assumed a homeostasis of the firing rate [59], [3], in the sense that if neuronal activity increases beyond an a-priori chosen set-point, synaptic contacts are pruned to decrease the excitatory drive, and the reverse for activity below the set-point. Indeed the correlation dependent structural plasticity model [27] shows this behavior. We have investigated whether our models show firing rate homeostasis by computing how the expected number of active contacts changes with the firing rate. This dependency can be chosen arbitrarily by adjusting a free parameter of the model (see Fig. 4***b***). Our model hence is capable of providing the proposed firing-rate homeostasis for properly chosen parameters.

To obtain a simple Markov process, we used the discrete categories “unrealized”, “inactive” and “active” to describe the state of a synaptic contact. Technically our model is similar to the cascade synapse model of [60] but adds the morphological interpretation of the synaptic states. The inactive contact might be closely related to silent synapses, but in the actual biological system such a clear-cut distinction between functional states can probably not be made, see for example [38]. Busetto et al. [61] found that silent synapses are abundant in the developing animal but vanish in the adult. However, only spines that were morphologically mature were included in their study, making no claim about existence of thin spines with small heads. Quantal EPSC analysis in the adult neocortex showed that close to all synaptic contacts of the connection L4–L23 are functional [55]. Our model of this connection also shows no inactive synapses in expectation, which renders them unobservable in practice. Further [62] find in cultured hippocampal slices that newly formed spines contain AMPA receptors. Small spines, however, are generally easy to miss, since they are often smaller than the resolution limit of optical microscopy [10], [61], and they may also be pruned again quickly after formation [63]. After all there is ample evidence that newly formed spines are small [21] and that AMPA receptor density correlates with volume [22]. We thus follow [43] and approximate thin, small spines as inactive synaptic contacts, and large spines as active ones as described above in detail.

As a consequence of the coarse-grained description of the state of synaptic contacts, all active synaptic contacts in our model produce an EPSP of a fixed amplitude 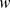. However, in biology this amplitude varies from contact to contact. Including a fine grained description of synaptic amplitudes in a structurally similar model as the one presented here would result in a massive increase of the dimension of the state space and is therefore potentially unfeasible. Such a dispersion of synaptic amplitudes would result in a different functional dependence of the mean (2) and variance (3) of the correlation trace 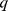 on the number of active contacts 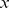. However, at a given synapse the mean would still be monotonically increasing with 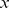. On a population level, the dispersion of synaptic amplitudes thus results in an additional contribution to the width of the distribution of the correlation trace 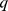 in (3). We can think of part of the noise 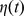 added to 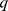 as representing this contribution. This reduces the precision of correlation detection at the dendritic spine. In a model with dispersion of synaptic amplitudes, we therefore expect to find qualitatively similar fits for our coarse grained model at a correspondingly reduced additional noise.

We defined that inactive synaptic contacts host NMDA receptors. The conductance of NMDA receptors increases upon a postsynaptic depolarization if the magnesium block is removed. At negative voltages NMDA channels have a smaller but non-vanishing conductance and hence mediate excitatory postsynaptic currents (EPSC). However, the time scale of NMDA activation is much slower than that of AMPA channels. A postsynaptic action potential partially caused by NMDA currents of one synaptic contact would thus occur much later than the presynaptic glutamate release. The postsynaptic depolarization is therefore less efficient in opening the NMDA receptors at another synaptic contact of the same synapse. This, however, is the crucial mechanism that allows correlation detection and cooperation in our model. Hence one may assume that NMDA currents contribute much less to the correlation trace, and thus have vanishing impact on the cooperative plasticity of our model. We therefore use the term “inactive” here in a functional sense.

In neonatal rat hippocampus also presynaptically silent synapses have been observed, which show a very low probability of transmitter release [64], [65]. However, even a low probability of release enables the formation of a postsynaptic correlation trace at the dendritic spine as in our model. Moreover, even presynaptic changes of the transmitter release have been reported to depend on such a correlation trace in a similar way [54]. The dependence of maturation and shrinkage/pruning on the correlation trace that we use here is a sufficiently generic model to also include these presynaptic mechanisms, although we do not intend to model them here explicitly.

The term structural plasticity describes a broad range of phenomena, many of which have not been addressed here. Competition between synapses from distinct neurons to a common postsynaptic neuron has been shown to be important for the emergence of cortical network structure [66]. In the more detailed models of structural plasticity in neuronal networks based on the activity of CaMKII [27], [67], cooperation and competition between synaptic contacts necessarily occurs. Here we assumed that synapses between different pairs of neurons develop independently, so inter-synaptic competition effects were not considered. Furthermore, structural plasticity also includes changes to the network structure that can come about by migration of axons on much longer time scales. Our model rather describes the steady state of the adult cortex, during which spines form and retract, but the axonal arborization can be assumed to be constant [13]. In lesion studies it has been shown that the steady state can become unstable and axons again begin to migrate [68].

Although simple and abstract in its description of complex cellular phenomena, our model can explain the cooperation of synaptic contacts in the adult neocortex, postulated in [20]. The model shows how continuously active structural plasticity can lead to the global configuration of synaptic contact numbers that was observed experimentally. The key ingredient of the model which mediates the necessary cooperation is a trace of the spike-timing correlations of the pre- and postsynaptic neuron. The resulting synaptic learning rule is local (it solely requires mechanisms at the synaptic contacts) but can nonetheless explain cooperative synapse formation.
